# Textbook oncologic outcomes in elderly patients undergoing neoadjuvant chemoradiotherapy and surgery for locally advanced rectal cancer: a multicenter study

**DOI:** 10.1007/s13304-025-02374-z

**Published:** 2025-08-25

**Authors:** Alessandra Pulvirenti, Carlotta Parati, Simona Deidda, Daniela Rega, Gino Guarino, Mirko Armas, Ilaria Govoni, Silvia Negro, Quoc Riccardo Bao, Paolo Delrio, Angelo Restivo, Gaya Spolverato

**Affiliations:** 1 Department of Surgical, Oncological and Gastroenterological Sciences (DiSCOG), University of Padua, Via Giustiniani 2, 35010 Padua, Italy; 2 Unit of General Surgery 3, University Hospital of Padua, Padua, Italy; 3 Department of Surgery, Colorectal Surgery Center, University of Cagliari, Cagliari, Italy; 4 Colorectal Surgical Oncology, Department of Abdominal Oncology, Istituto Nazionale Tumori-IRCCS “Fondazione G. Pascale”, Naples, Italy

**Keywords:** Locally advanced rectal cancer, Textbook oncological outcomes, Elderly, Neaoadjuvant therapy

## Abstract

Standard treatment of locally advanced rectal cancer (LARC) involves neoadjuvant chemoradiotherapy (nCRT) followed by total mesorectal excision (TME). However, in older patients, comorbidities and frailty may limit the feasibility of this approach. This study evaluates the incidence of Textbook Oncological Outcome (TOO), a composite quality metric in surgical oncology, in patients aged ≥ 70 years undergoing low anterior resection (LAR) for LARC after nCRT and identifies predictors of achieving this benchmark. We retrospectively analyzed 157 elderly patients with LARC treated by nCRT followed by LAR between 2011 and 2023 in three Italian tertiary centers. TOO was defined as R0 resection, no prolonged hospital stays (LOS < 11 days), no major complications (Clavien-Dindo < 3b), no 30-day readmissions, and no 90-day mortality. TOO was achieved in 61% (*n* = 95) of patients. The most common limiting factor was prolonged LOS, met by 70.7% (*n* = 111), followed by the absence of 30-day readmission (87%, *n* = 137). The most consistently achieved components were no major complications (91%, *n* = 143), R0 resection (98%, *n* = 154), and no 90-day mortality (100%). Univariate analysis revealed that a lower (y)pTNM stage was the only significant predictor of TOO (*p* = 0.008), whereas age and surgical approach were not significantly associated. Survival and recurrence did not differ between patients who did and did not achieve TOO. TOO was achieved in a significant proportion of elderly patients undergoing LAR for LARC after nCRT. A multimodal approach is feasible and effective even in elderly.

**Supplementary Information:**

The online version contains supplementary material available at 10.1007/s13304-025-02374-z.

## Introduction

Colorectal cancer (CRC) is the third most diagnosed malignancy and the second leading cause of cancer-related mortality worldwide [1]. The incidence of CRC is rising in the elderly population, with over half of newly diagnosed cases occurring in individuals aged over 70 years [2, 3]. Among these, approximately 35% are rectal cancers, the majority of which present as locally advanced disease at diagnosis [4]. For patients with locally advanced rectal cancer (LARC) the standard treatment consists of neoadjuvant chemoradiotherapy (nCRT) followed by total mesorectal excision (TME), a strategy that has significantly improved oncologic outcomes [5]. However, in elderly patients, the implementation of this multimodal approach is often limited due to a higher prevalence of comorbidities, reduced performance status, and frailty, leading to a greater risk of undertreatment [6–9]. As a result, older patients with rectal cancer frequently experience lower rates of guideline-concordant care and have poorer cancer-specific survival [8, 10]. Optimizing oncologic outcomes in this vulnerable population while minimizing treatment-related morbidity remains a clinical challenge.

Recently, Textbook Oncologic Outcome (TOO) has emerged as a composite quality metric in surgical oncology, integrating traditional surgical benchmarks, such as postoperative morbidity and mortality, with key oncologic indicators, including negative resection margins, adequate lymphadenectomy, and adherence to neoadjuvant and adjuvant treatment protocols. TOO provides a standardized measure to assess the quality and completeness of multimodal oncologic treatment. TOO is particularly relevant in elderly patients, where achieving an optimal balance between effective cancer control and treatment-related toxicity is crucial. Although the TOO framework has been applied to colorectal cancer in a limited number of studies, its feasibility and prognostic significance in elderly patients undergoing surgery for LARC remain underexplored [11]. Understanding the pre-treatment factors associated with TOO achievement in this population is critical for refining treatment strategies and improving outcomes. This study aimed to evaluate the incidence of TOO in patients aged ≥ 70 years who underwent surgery for LARC following nCRT at three high-volume Italian tertiary centers and to identify pre-treatment predictors associated with achieving this benchmark. By defining factors that influence TOO attainment, this study provides insights into optimizing oncologic care and improving surgical outcomes in elderly patients with rectal cancer.

## Method

### Patients population and data collection

Prospectively maintained databases from three high-volume Italian centers (University Hospital of Padua, University Hospital of Cagliari, and Istituto Nazionale Tumori-IRCCS Fondazione G. Pascale, Naples) were queried to identify patients aged ≥ 70 years who underwent curative-intent surgery for locally advanced mid-to-lower rectal cancer between 2011 and 2023. Inclusion criteria encompassed patients who underwent low anterior resection (LAR) following nCRT for histologically confirmed adenocarcinoma. Patients were excluded if metastatic disease was identified at preoperative staging or intraoperatively, or if they underwent abdominoperineal resection or pelvic exenteration. Variables of interest included patient demographics, clinical characteristics, pathological findings, details of surgical and neoadjuvant treatments, and surgical and oncologic outcomes. Locally advanced disease was defined as clinical stage cT3-4 and/or cN + based on CT and MRI findings at diagnosis, in accordance with the AJCC 8th edition staging system. Mid-to-lower rectal cancer was defined as a tumor located ≤ 10 cm from the anal verge on preoperative MRI. The surgical approach was determined according to the intention-to-treat principle. The study was approved by the Institutional Review Board at each participating institution.

### Textbook oncological outcomes

To date, no universally standardized definition of TOO in rectal cancer exists. Therefore, we adopted the criteria for colorectal surgery as established by an Italian expert consensus [12]. The TOO was defined as meeting all of the following five criteria: (1) a radical (R0) resection with negative longitudinal and circumferential margins; (2) a length of stay (LOS) that does not exceed the 75th percentile of the current cohort; (3) the absence of major postoperative complications, defined as Clavien-Dindo grade < 3b; (4) no hospital readmission within 30 days post-surgery; and (5) no 90-day mortality. Given that all included patients received nCRT, oncologic treatment was not incorporated into the TOO definition. Moreover, lymph node yield was excluded, as no consensus exists on the optimal number of harvested nodes in post-nCRT specimens due to the well-documented reduction in nodal retrieval following neoadjuvant therapy [5, 13].

### Statistical analysis

Disease and treatment characteristics were summarized using median and interquartile range for continuous variables, and frequency and percentages for categorical variables. The Fisher exact test and Wilcoxon rank-sum test for categorical and continuous variables, respectively, were used to compare subgroups. Overall Survival (OS) was calculated from the date of curative surgery until the date of death and estimated using Kaplan–Meier methods. Patients alive at the time of the study were censored at the date of the last follow-up. Cumulative incidence of recurrence was estimated using the competing risks method. Patients who died without a recurrence were censored at the date of death. All analyses were performed in R (R Foundation for Statistical Computing, Vienna, Austria). P values were 2-sided and were considered statistically significant if < 0.05.

## Results

### Study population

A total of 157 elderly patients with LARC treated with nCRT were included. Detailed patient characteristics are provided in Table 1. The median age at diagnosis was 75 years (IQR, 72–78). At pretreatment evaluation, 7 (4.5%) patients were staged as cT2, 118 (76%) as cT3 and 30 (19%) as cT4. Nodal involvement was detected at diagnosis in 138 (88%) patients, and the median tumor distance from the anal verge was 7.0 cm (IQR, 5.0–9.0).

**Table 1:** Study Population Clinical Characteristic

Characteristic	N = 157^1^
Female	55 (35%)
Age at Surgery, years	75 (72–78)
ASA	
1–2	87 (58%)
3–4	64 (42%)
Body Mass Index, Kg/m^2^	24.8 (22.4–27.6)
Pretreatment CEA, ng/mL	2 (1–4)
cT stage	
cT2	7 (4.5%)
cT3	118 (76%)
cT4	30 (19%)
cN stage	
cN-negative	18 (12%)
cN-positive	138 (88%)
Tumor distance from anal verge, cm	7.00 (5.00–9.00)

^1^n (%); Median (Q1-Q3)

### Surgical outcomes and pathology

All patients underwent LAR, with a minimally invasive approach employed in 91 (58%) cases. Of these, 7 (6.2%) procedures required conversion to open surgery. Ostomy creation was performed in nearly all patients (*n* = 153, 99%), with 148 (96.7%) receiving an ileostomy and 5 (3.3%) a colostomy. Ileostomy reversal was achieved in 77% of cases (*n* = 118), with a median time to reversal of 174 days (IQR 87–263). The median LOS was 8 days (IQR, 7–11). Severe complications (Clavien-Dindo grade ≥ 3b) occurred in 14 (8.9%) patients, and there was no 90-day mortality. An R0 resection was achieved in 154 (98%) patients, with a median of 13 harvested lymph nodes (IQR, 8–17). A pathological complete response (pCR) was observed in 25 cases (16%, Table 2).

**Table 2:** Surgical Outcomes and Pathology

Characteristic	N = 157^*1*^
Minimally-invasive	91 (58%)
Clavien-Dindo ≥ 3b	14 (8.9%)
Reintervention	12 (7.6%)
Length of stay, days	8 (7–11)
Readmission within 30 days	20 (13%)
No. of harvested lymph nodes	13 (8–17)
R status	
R0	154 (98%)
R1	3 (1.9%)
(y)pT stage	
(y)pT0	25 (16%)
(y)pT1	15 (9.6%)
(y)pT2	53 (34%)
(y)pT3	62 (39%)
(y)pT4	2 (1.3%)
(y)pN stage	
(y)pN0	116 (77%)
(y)pN1	22 (15%)
(y)pN2	13 (8.6%)
(y)pStage	
(y)pStage 0-I	84 (54%)
(y)pStage II	38 (24%)
(y)pStage III	35 (22%)

^1^n (%); Median (Q1-Q3)

### Achievement of textbook oncological outcome

**Fig. 1:**
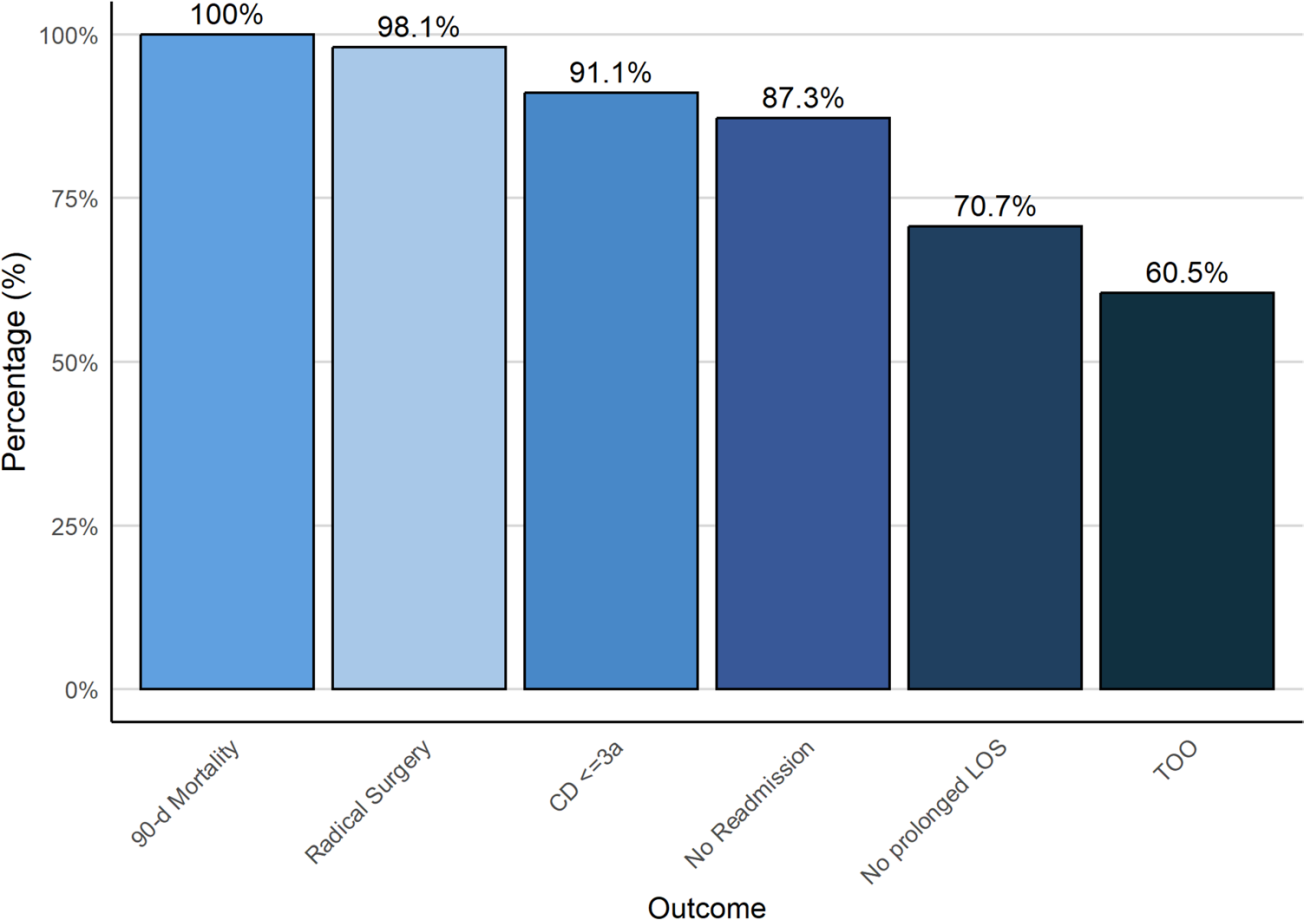
Proportion of Patient Achieving TOO and Each TOO Component

Overall, 61% of patients (*n* = 95) met all the criteria for a TOO. Among the individual components, a LOS of less than 11 days was the most frequently unmet, achieved by only 111 patients (70.7%) (Fig. 1). The absence of readmission within 30 days was the second most restrictive criterion, met by 137 patients (87%). Among the 20 readmitted patients, the most common cause was acute kidney injury, which occurred in six cases, followed by anastomotic leaks and abdominal fluid collections, each reported in three cases. In contrast, the components most consistently achieved were the absence of 90-day mortality (100%), R0 resection (98%), and the absence of major postoperative complications (Clavien-Dindo ≤ 3a in 91%). Univariate analysis identified (y)pTNM stage as the only significant predictor of achieving TOO (*p* = 0.008) (Table 3). Compared with patients who achieved a pathological complete response (pCR) or stage I disease, those with stage II and stage III disease were progressively less likely to meet TOO criteria, with odds ratios of 0.60 (95% CI: 0.27–1.135) and 0.33 (95% CI: 0.15–0.74), respectively. Neither age at surgery nor the surgical approach significantly impacted on the likelihood of achieving TOO.

**Table 3:** Factors associated with TOO achievement

Characteristic	N	OR^1^	95% CI^1^	p-value
Age at Surgery, yrs	157	0.95	0.88, 1.04	0.26
ASA	151			0.12
1–2		–	–	
3–4		0.60	0.31, 1.15	
Body Mass Index	154	0.99	0.92, 1.07	0.84
Pretreatment CEA	138	0.99	0.95, 1.00	0.12
Anal Verge Distance, cm	157	0.94	0.82, 1.09	0.42
Surgical Approach	157			0.49
Minimally invasive		–	–	
Open		1.25	0.66, 2.42	
Converted to Open	113			0.83
No		–	–	
Yes		0.84	0.18, 4.45	
(y)pStage	157			0.008
(y)pStage 0-I		–	–	
(y)pStage II		0.58	0.26, 1.30	
(y)pStage III		0.28	0.12, 0.64	

^1^OR, Odds Ratio; CI, Confidence Interval

### Oncological outcomes

**Fig. 2:**
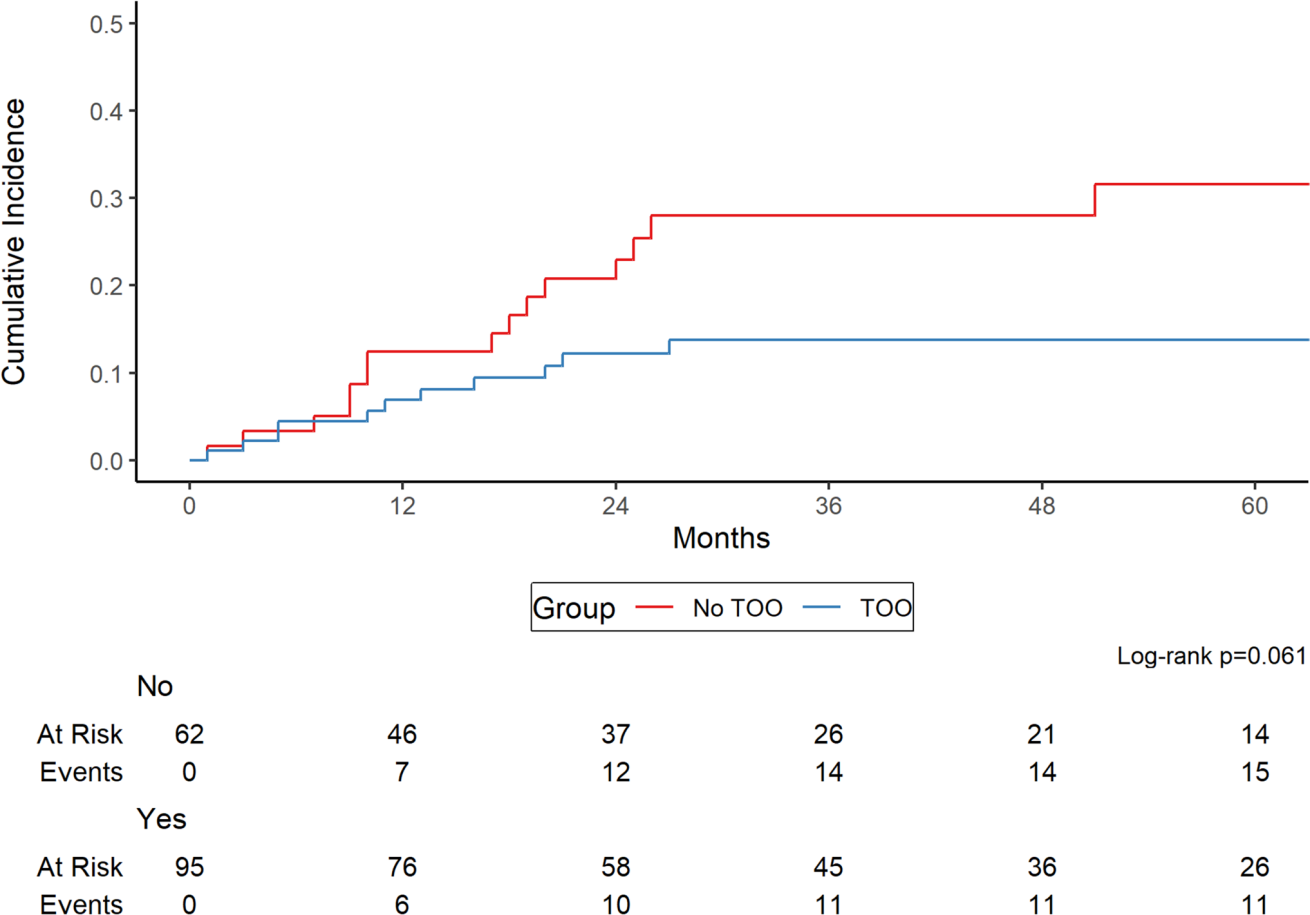
Cumulative Incidence of Recurrence According to TOO

The median follow-up among survivors was 34 months, during which 21 patients died. Overall survival for the entire cohort was 95%, 94%, and 85% at 2-, 3-, and 5-years post-surgery, respectively, with no significant difference between patients who achieved a textbook oncologic outcome (TOO) and those who did not (*p* = 0.13, Supplemental Content 1). Recurrence, whether distant or local, occurred in 27 patients at the time of the study. The rate of cumulative incidence of recurrence at 2-, 3- and 5-years was 17%, 19% and 21%. Although the recurrence rate was higher in patients who did not achieve TOO, this difference did not reach statistical significance (*p* = 0.061, Fig. 2).

## Discussion

Locally advanced rectal cancer presents significant treatment challenges, particularly in elderly patients with multiple comorbidities and reduced performance status. Standard nCRT followed by TME is often difficult to implement in this population. In this study, we evaluated the feasibility of achieving optimal oncological and surgical outcomes using the TOO metric in elderly patients.

Our multicenter study demonstrates that high-quality, multimodal treatment for LARC is possible in elderly patients at high-volume tertiary centers. In our cohort, 60% of patients achieved the TOO, underscoring that optimal surgical and oncologic benchmarks can be reached even in this high-risk group [14]. Although nearly all patients underwent a successful radical resection, prolonged LOS emerged as the primary barrier to a complete TOO, with readmissions serving as an additional challenge. Notably, in this study, the median LOS was 8 days, and a prolonged hospital stay was defined as exceeding 11 days, a more stringent criterion than the LOS of up to 14 days typically deemed acceptable in most rectal cancer studies [11, 14, 15]. These findings are particularly relevant, as nCRT has been previously identified as a limiting factor for achieving optimal surgical outcomes, irrespective of patient age [14]. Consequently, our data support the inclusion of elderly patients in standard multimodal neoadjuvant treatment protocols, provided they are managed in specialized tertiary centers.

In assessing predictors of TOO, we observed that factors traditionally presumed to impact outcomes, such as age, the choice of surgical approach, and conversion to open surgery, were not significantly associated with meeting TOO criteria [14]. Instead, the post-treatment pathological stage emerged as the key determinant; patients achieving a pathological complete response or stage I disease were significantly more likely to meet TOO criteria compared with those with stage II or III disease. This finding suggests that, similarly in younger patients, the local tumor extension and the complexity of surgical resection play a critical role in achieving optimal perioperative outcomes. However, these patients can be identified during post-nCRT restaging and may benefit from tailored preoperative optimization strategies to improve their baseline performance status, thereby enhancing their likelihood of achieving TOO.

Our analysis highlights distinct differences in the surgical management of LARC in elderly patients compared with younger cohorts described in the literature [14]. In this study, minimally invasive techniques were employed less frequently, and nearly all patients underwent ostomy creation. This is consistent with previous studies, showing that elderly patients with LARC are treated with open surgery in up to 87% of cases, and receive a temporary or permanent ostomy in 94% of cases [8, 16]. This more conservative approach likely reflects the increased frailty and comorbidities in elderly, which may limit the feasibility of laparoscopic or robotic procedures and necessitate protective measures, such as ostomy creation, to mitigate postoperative complications. Notably, despite the routine use of diversion ostomies, overall outcomes remained satisfactory, with a reversal rate of 70% and a low incidence of ostomy-related complications. This is especially relevant, as both older age and the use of nCRT are well-established risk factors that prevent temporary stoma reversal [17].

Oncologic outcomes in our cohort were similar to those reported in major nCRT trials, with 5-year overall survival and recurrence rates aligning with broader population data [16, 18, 19]. Although post-surgical complications are known to adversely affect oncologic outcomes, we found no significant differences in survival or recurrence between patients who achieved TOO and those who did not [20, 21]. While there was a trend toward a lower cumulative incidence of recurrence in patients achieving TOO, this observation was confounded by a higher prevalence of less advanced disease in this group. Consequently, while TOO is indicative of excellent surgical quality, its achievement does not necessarily translate into reduced relapse risk or improved overall survival, as radical surgery and minimal postoperative complications were achieved irrespective of TOO status. However, given the size of our cohort and the low number of events, larger studies are needed to clarify whether TOO can serve as a prognostic indicator of superior oncologic outcomes.

This study has several limitations. Its retrospective design and the selective inclusion of patients deemed fit for nCRT —who consequently have a better performance status—may limit the generalizability of our findings to the broader elderly rectal cancer population. Furthermore, the absence of a standardized definition for TOO in rectal cancer poses a significant challenge and difficulties in comparing results across different studies. Although an Italian consensus has outlined desirable outcomes those were for colorectal cancers and not specific for LARC [12]. As a result, these criteria were not fully applicable to patients undergoing nCRT. Finally, the use of LOS as a quality indicator is contentious, as LOS can be affected by numerous factors unrelated to surgical quality, including cultural practices, healthcare facility type (private vs. public), outpatient follow-up protocols, and social issues impacting discharge.

In conclusion, our study shows that standard nCRT followed by surgery is feasible with optimal surgical and oncological outcomes in elderly patients with LARC treated in a referral center. Further larger studies are warranted to investigate the prognostic value of TOO and to refine treatment strategies for this patient population.

## Supplementary Information

Below is the link to the electronic supplementary material.

Supplementary file1 (DOCX 56 KB)

## Data Availability

The datasets generated and/or analyzed during the current study are available from the corresponding author on reasonable request.
