# Supplementary figures and images for: Textbook oncologic outcomes in elderly patients undergoing neoadjuvant chemoradiotherapy and surgery for locally advanced rectal cancer: a multicenter study
Source: Updates Surg. 2025 Aug 25;77(8):2285–91. doi: 10.1007/s13304-025-02374-z (PMC12630306; doi:10.1007/s13304-025-02374-z)

**Supplemental Contents**

Supplemental Content 1. Overall Survival According to TOO


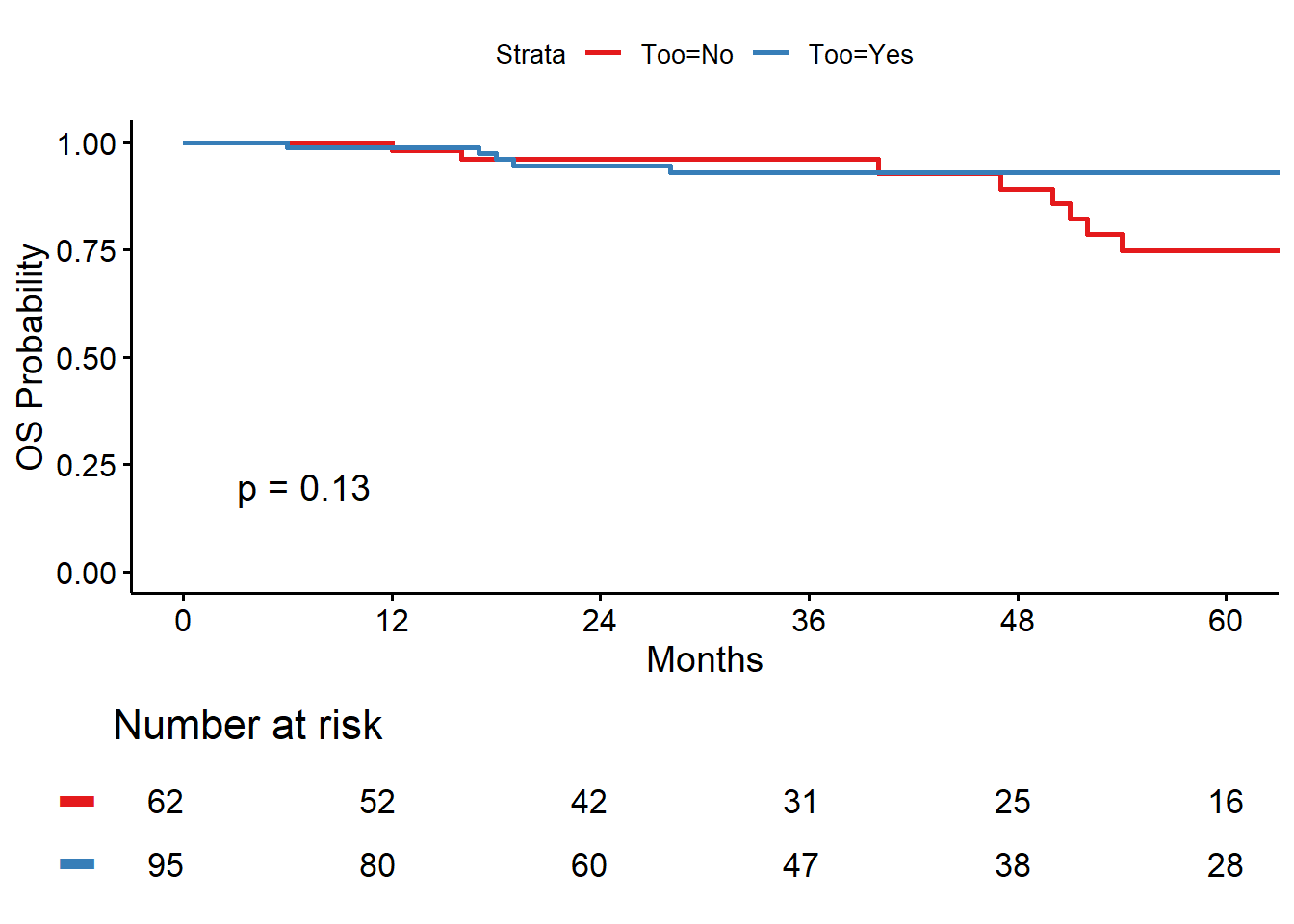

Supplement: Supplementary file 1 — Supplementary file1 (DOCX 56 KB) [file 13304_2025_2374_MOESM1_ESM.docx]
